# The role of social support in overcoming leisure constraints in recreational sport participation

**DOI:** 10.3389/fspor.2025.1646511

**Published:** 2025-08-14

**Authors:** Apostolia Ntovoli, Georgina Stavropoulou, Thomas Karagiorgos, Yannis Lianopoulos, Garyfallos Anagnostou, Elena Papacosta, Kostas Alexandris

**Affiliations:** ^1^ School of Health Sciences, Department of Life and Health Sciences, Frederick University, Limassol, Cyprus; ^2^ Laboratory of Management of Sports, Recreation, and Tourism, School of Physical Education and Sport Science, Aristotle University of Thessaloniki, Thessaloniki, Greece; ^3^ Sport Entrepreneurship and Innovation Lab, Department: School of Physical Education & Sport Science (Serres), Aristotle University of Thessaloniki, Thessaloniki, Greece

**Keywords:** social support, leisure constraints, barriers, recreational sport participation, leisure negotiation

## Abstract

**Introduction:**

Following the hierarchical model of leisure constraints and the negotiation proposition, this study aimed to investigate whether individuals with different levels of leisure constraints exhibit varying scores in their perceived social support. By testing the relationship between constraints and social support, the study contributes to the literature by adding one more factor that determines the successful negotiation of leisure constraints.

**Methods:**

The data were collected by an online survey with a convenient sample of the adult Greek population. The leisure constraint and the social support questionnaires were used to collect the data.

**Results:**

The cluster analysis revealed three groups with different leisure constraint scores. The group with the lowest constraint scores had the highest social support scores, while the group with the highest interpersonal scores had the lowest social support scores.

**Discussion:**

These results further extend the hierarchical model of leisure constraints, showing that social support is one of the factors that should be included within the negotiation proposition. They interact with leisure constraints and determine their successful negotiation.

## 1 Introduction

Physical inactivity is now recognized as a widespread and escalating global concern. According to the World Health Organization (WHO) (1), approximately 1.4 billion adults worldwide, which is equivalent to 27.5% of the global adult population, fail to meet the recommended levels of physical activity, which is necessary for maintaining and enhancing physical and mental health. These low participation rates are of particular concern given the established relationship between exercise and reduced risk of chronic diseases (2–6). Considering the notably low participation in sports and physical activities, it is essential to examine key predictors of exercise participation, such as leisure constraints and the factors that interact with them (7, 8).

Research on leisure constraints has been a topic widely explored in academic literature over the past three decades, owing to both its theoretical significance and practical implications (7–11). From a theoretical standpoint, the introduction of the hierarchical model of leisure constraints (7, 12) and subsequent negotiation propositions (13–15) has contributed to a deeper understanding of how individuals make decisions regarding participation in sports, leisure, and recreational activities (16, 17). From an applied perspective, research on leisure constraints provides valuable insights for practitioners, enabling the development of strategies and policies aimed at reducing barriers and promoting greater engagement in sports and physical activity (13, 16, 18).

Upon introducing the leisure negotiation proposition, Jackson (19) proposed that several intrinsic and extrinsic factors for an individual may interact with perceptions of constraints to determine their successful negotiation. Factors that have been proposed and tested include motivation (8, 20), attitudes (3, 7, 8), and personality (21, 22). One of the factors that has been proposed to positively influence individuals' decisions to start taking part in sports and get committed to sport participation (7, 19) but has not been empirically tested in relation to the perception of leisure constraints, is social support (7, 19). Some evidence for this positive relationship was provided in the study by Chen et al. (23), who, however, examined the role of social support in relation to the seniors' travel constraints, negotiation strategy, and travel intentions.

Research has indicated that support from family and friends, commonly referred to as “significant others”, could help individuals to negotiate leisure constraints and overcome them, leading to positive behavioral outcomes (24–27). As previously noted, the role of social support as a factor that might interact with leisure constraints and determine their individual successful negotiation has not been empirically examined so far. Filling this research gap in the current study, we examined if and how social support interacts with the perception of leisure constraints, as defined by the hierarchical model of leisure constraints, which was used as the theoretical framework of the study. Jackson et al. (19) proposed that various factors, including attitudes, perceptions, and motivation, may serve as moderating variables in the relationship between leisure preferences, constraints, and participation. Effective negotiation, facilitated by one or more of these factors, can lead to either full or modified participation in recreational activities. Conversely, when negotiation efforts are unsuccessful, constraints may act as barriers, blocking participation. In the present study, we argue that social support may play an important role in mitigating perceived constraints, ultimately influencing an individual's intentions to engage in recreational sports.

We used the term “recreational sports” to describe sports and exercise activities undertaken by individuals during their leisure time, aligning with definitions provided in previous research (28–30). This term offers a more specific focus compared to the broader concept of “physical activity”, as defined by the WHO (1). Physical activity encompasses all forms of movement, including those performed during leisure time, for transportation purposes, or as part of occupational and domestic responsibilities.

## 2 Theoretical background

### 2.1 Leisure constraints

Jackson (19) defined constraints as “factors that researchers assume, and individuals perceive or experience, as limitations that restrict the development of leisure preferences and hinder or entirely prevent participation in leisure activities” (p. 279). Crawford and Godbey (31) categorized these constraints into three distinct types: structural, interpersonal, and intrapersonal. Structural constraints encompass external factors such as time limitations, lack of resources, and deficiencies in facilities or services. Interpersonal constraints pertain to the absence of partners with whom to engage in sport or exercise (32). Finally, intrapersonal constraints are internal and include psychological barriers such as low self-esteem, perceived lack of ability, and societal values (9, 33). These three categories of constraints were integrated into a hierarchical model of leisure decision-making by Crawford et al. (12), based on their influence on leisure preferences and actual participation. Among these, intrapersonal constraints represent the most significant barriers to exercise participation, blocking it, while structural constraints exert a lesser impact, primarily leading to modifications rather than complete avoidance of participation. Interpersonal constraints, however, have the potential to both block and modify participation (12, 32). One of the developments of the leisure constraint theory has been the introduction of the negotiation and balance propositions (23). Jackson et al. (13) proposed that the successful negotiation of leisure constraints determines the influence of constraints on leisure participation. In this line, for some individuals, constraints act as blocking factors (no participation), while for some others, they act as limiting factors. In the second case, the outcome of the influence of leisure constraints can be reduced participation or participation in alternative activities. This means that some individuals might successfully negotiate and overcome constraints, while others do not. This successful negotiation is determined by the strength of constraints in relation to factors that facilitate participation. Examples of these factors that have been proposed in the literature are motivation, personality, and social interaction (8, 20, 25, 34). While there is empirical evidence on the interaction between constraints with motivation, and personality, the influence of social support on the successful negotiation of leisure constraints has not yet been empirically tested.

### 2.2 Social support

A variety of terms and classifications have been employed to define social support and its related constructs (35–39). Heaney and Israel (40, p. 191) defined it as “aid and assistance exchanged through social relationships and interpersonal transactions”. Social support embodies the idea that supportive social actions or the perception of their availability can be used by individuals when needed. Therefore, a distinction exists between the actual support that an individual might receive and the perceived one. Many scholars do not clearly differentiate between perceived and actual support. However, perceived support is often more consistently associated with positive health outcomes and sport participation (38, 39).

Social support can be classified into various types and sources. The primary types of support include instrumental support (e.g., tangible financial or material assistance), appraisal support (e.g., companionship and social comparison), informational support (e.g., provision of advice or resources), and emotional support (e.g., expressions of empathy, concern, encouragement, or nurturance) (39, 40). Schaefer et al. (41) proposed a more detailed structure with five distinct types: (a) emotional support, (b) esteem support, (c) network support, (d) informational support, and (e) tangible or instrumental support.

Emotional support refers to the assistance provided to meet an individual's emotional needs and is the most referenced type of social support in the literature. Esteem support involves actions aimed at enhancing an individual's self-confidence and reinforcing their belief in their ability to cope with challenges or achieve their personal goals. Network support pertains to an individual's perception of the availability of a supportive social network, indicating whether there are significant others who can offer various forms of assistance. Informational support relates to the availability and provision of relevant information from significant others, enabling individuals to make informed decisions on personal matters, such as selecting appropriate facilities for physical activity, identifying high-quality fitness centers, or finding parks that offer outdoor exercise opportunities. Finally, tangible or instrumental support refers to practical assistance provided to an individual to help manage daily responsibilities, such as childcare or transportation (35, 42, 43).

The key sources of support are primarily identified as family, friends/peers, and significant others (39, 40), with these relationships collectively forming social support networks (44). The characteristics of such networks can influence both the quantity and quality of social support available. Social support networks differ based on attributes such as size, frequency of interactions, depth of relationships, and the level of homogeneity among social connections (45, 46).

### 2.3 Social support, physical activity, and leisure constraints

Social support has been linked to various behavioral outcomes, including dietary habits and engagement in Physical Activity (PA) (47, 48). Specifically, Tamers et al. found that higher perceived social support in the workplace is related to increased levels of PA as well as greater consumption of fruits and vegetables. Furthermore, studies have shown a positive relationship between social support and PA participation, particularly among women (49, 50–53). Ayotte et al. (49) showed the impact of perceived social support from family members, specifically companionship, informational, and esteem support, on PA engagement. Similarly, Bauman et al. (50) investigated the social determinants and correlations of PA participation, concluding that family and parental support were positively associated with PA engagement, particularly in high-income households.

In conclusion, social support for physical activity encompasses encouragement, role modeling, or other facilitative actions provided by an individual's social network (54, 55). Social support has been directly associated with increased physical activity levels across various populations (56), including different racial and ethnic groups (47–59).

Given the significance of social support in the decision-making process regarding sports participation, we argue that, from a theoretical and applied perspective, it is important to test the influence of social support on the perceptions of leisure constraints, using the hierarchical model of leisure constraints (19) as a theoretical framework. The model proposes that intrapersonal constraints are the most powerful determinants of sport participation, as they influence preferences that are difficult to overcome. On the other hand, structural constraints, like external ones, are the least powerful and the easiest to overcome. Finally, interpersonal constraints influence both preferences and actual behavior (participation). As previously noted, it has been proposed that some individuals are capable of overcoming leisure constraints and engaging in sport participation. This is a result of a successful negotiation of leisure constraints (7, 8, 15). We propose that social support can be one of the factors that interact with the perception of leisure constraints and act as facilitators of overcoming them. Either propositions are based on the definition of negotiation strategies as either cognitive or behavioral. Jackson and Rucks (60) proposed, by combining qualitative and quantitative methods, that cognitive strategies often involved reframing constraints (e.g., rationalizing limited access), while behavioral strategies included tangible adjustments such as rearranging time commitments, developing necessary skills, altering social networks, or recalibrating leisure goals. We argue that instrumental and informational support can serve as behavioral negotiation strategies, while emotional support can function as cognitive strategies. Jackson and Rucks (60) reported that behavioral strategies were more effective in overcoming leisure constraints. Considering that social support can positively influence individuals' motivation to engage in sports, which is a strong indicator of the successful negotiation of leisure constraints (7–9), the current study aimed to test if individuals with different levels and nature of leisure constraints report different social support levels. In this line, the research objectives of the paper were set as clearly as possible:

• To cluster individuals based on their scores on interpersonal, intrapersonal, and structural constraints.
• To compare these constraint cluster groups' scores in terms of their social support scores and discuss the results with reference to the hierarchical model of leisure constraints (12) and the leisure negotiation proposition (19).

## 3 Methodology

The data was collected with an online questionnaire, which was posted on the social media of the research team, inviting people to fill out the questionnaire. The goal was to draw a sample of the adult general population, large enough to run the statistical analysis required in order to test the theoretical model. It has to be noted that our goal was not to have a representative sample of the general population of the country. With this process, we achieved a sample of three hundred and eighty two (*N* = 382) adult individuals. Informed consent was obtained from all participants before completing the questionnaire. Certain limitations associated with the sampling method must be acknowledged. The study utilized a convenient sampling method. A convenient sample is always subject to sampling error; subsequently, the findings cannot be generalized. However, this sample was judged satisfactory to statistically test the theoretical model of the study. The questionnaire included self-reported measures of recreational sport participation, constraints on participation, and social support. Recreational sports were defined as sports and exercise activities performed during leisure time (7, 20). To ensure clarity, a list of sport activities—including walking for exercise—was provided, based on previous research [e.g. (20, 61)].

Concerning gender, the sample comprises 45.8% male and 54.2% female participants. Regarding the age of the participants, the data indicated that 35.8% of the sample fell within the 31–45 age group, 29.2% were between 46 and 55 years of age, 18.7% were between 18 and 30 years of age, and 16.3% were 55 years of age or older. In terms of educational attainment, the majority of respondents attended secondary school (35.3%), followed by university graduates (25.5%). A smaller percentage of the population has completed primary education (9.5%), some of them attended a Technological Educational Institute (11.3%), and some have earned a Master's degree (11.3%), or have had Vocational Training (7.1%). A comparatively small percentage of the participants (19.9%) reported engagement in sports during the previous four weeks, while a substantial majority (80.1%) reported non-participation. Of the participants, 16.2% indicated that they engaged in recreational sports daily, while 41.9% stated that they participated three to five times per week. It was reported by a smaller proportion of the sample that they engaged in sporting activities one to two times per week (32.4%) or less than once per week (9.5%). The demographic and behavioral profile of the sample is presented in Table 1.

**Table 1:** Demographic characteristics of gender, age, educational level, participation in sports, and frequency of participation.

Gender	Frequency	Percent
Men	174	45.8
Women	206	54.2
Total	380	100
Age	Frequency	Percent
18–30	71	18.7
31–45	136	35.8
46–55	111	29.2
>55	61	16.3
Total	379	100
Education	Frequency	Percent
Primary education	36	9.5
Secondary education	134	35.3
Public institute of vocational training	27	7.1
Technological educational institute	43	11.3
University	97	25.5
Master	43	11.3
Total	380	100
Sport participation	Frequency	Percent
Yes	76	19.9
No	306	80.1
Total	382	100.0
Frequency of sport participation	Frequency	Percent
Almost every day	12	16.2
3–5 times per week	31	41.9
1–2 times per week	25	32.4
Less than one time per week	8	9.5
Total	76	100

Leisure constraints were assessed using the scale developed by Alexandris and Carroll (28), which has been successfully validated in Greek populations and extensively documented in the literature (e.g., 7–8). This scale includes sixteen items categorized into three dimensions: structural constraints (six items), interpersonal constraints (three items), and intrapersonal constraints (seven items). Responses were measured on a five-point Likert scale ranging from “very important” to “not important”.

Social support was evaluated using a unidimensional ten-item scale. This was developed by Sallis et al. (52), as adapted from the original scale by Sallis et al. (62). A five-point Likert scale, ranging from “never” to “very often”, was used.

As noted, the measurement of these sub-dimensions was conducted using validated Likert-scale items. For each sub-dimension, mean scores of the relevant items were calculated to represent the composite score for each respondent, in line with prior research utilizing these scales. This approach enabled straightforward comparisons across dimensions, as higher mean values indicate greater perceived constraints (e.g., higher time scores reflect more significant time-related barriers). No cut-off criteria were applied; rather, the scores were interpreted on a continuum, with relative comparisons made between sub-dimensions and clusters

## 4 Statistical analysis

Initially, descriptive statistics were utilized in order to present frequencies for structural constraints (time, facilities, cost), intrapersonal constraints (psychological, lack of interest, previous experience), interpersonal constraints (lack of partners, lack of knowledge), and social support. The mean, standard deviation, mode, variance, skewness, kurtosis, minimum, and maximum are also displayed. K-Means cluster analysis was also employed to investigate groupings among individuals based on three constraint dimensions and their sub-dimensions: (a) structural constraints (sub-dimensions of time, facilities, cost), (b) intrapersonal constraints (sub-dimensions of psychological, knowledge, lack of interest, previous experience), and (c) interpersonal constraints (sub-dimensions of lack of partners, lack of knowledge). The measurement of these sub-dimensions was conducted using validated Likert-scale items, with the mean score of these items to represent each sub-dimension as a composite score per respondent. The optimal number of clusters (k) was identified through cluster centroids, within-cluster sum of squares, and Analysis of variance. The point of inflection was determined, and a k value of 3 groups was selected. The K-Means algorithm then partitioned the dataset into distinct clusters, with each cluster representing individuals with similar profiles of perceived constraints. An investigation was conducted into the cluster centroids to interpret the relative levels of structural, intrapersonal, and interpersonal constraints that characterized each group. The analysis of variance (one-way ANOVA) was employed to ascertain which variables contributed most significantly to the formation of clusters. Subsequent to the implementation of K-Means Cluster analysis, an analysis of variance (one-way Anova) was conducted with the objective of investigating whether the three clusters are differentiated in relation to social support. The Bonferroni correction was also employed to identify the differences among the three groups, as the data met the criterion of homogeneity of variances.

## 5 Results

Descriptive statistics were used for structural, intrapersonal, and interpersonal constraints and their sub-dimensions. Table 2 demonstrates that the constraint sub-dimensions reveal notable differences in the perceived intensity among the participants. In consideration of the structural constraints, it was found that Time (*M* = 4.3, sd = 1.50), Facilities (*M* = 3.8, sd = 1.54), and Cost (*M* = 4, sd = 1.73) all scored relatively highly. This finding indicates that these are commonly perceived barriers. Within the confines of intrapersonal constraints, Psychological Factors (*M* = 3.80, sd = 1.52) and Lack of Interest (*M* = 3.61, sd = 1.52) emerged as the most salient factors. Previous Experience exhibited the lowest means (*M* = 2.69, sd = 1.39), suggesting that it is less of a constraint for most individuals. Regarding interpersonal constraints, Lack of Partners (*M* = 3.24, sd = 1.68) was found to present moderate values and so does Lack of Knowledge (*M* = 3.13, sd = 1.65). Finally, the mean value of the Social Support variable was found to be low (*M* = 2.47, sd = .98).

**Table 2:** Descriptive statistics for structural constraints, intrapersonal constraints, interpersonal constraints, and social support.

Descriptive statistics	Structural constraints			Intrapersonal constraints			Interpersonal constraints		Social support
	Time	Facilities	Cost	Psychological	Lack of interest	Previous experience	Lack of partners	Lack of knowledge	
Mean	4.3	3.80	4	3.80	3.61	2.69	3.24	3.13	2.47
Std. deviation	1.50	1.54	1.73	1.52	1.52	1.39	1.68	1.65	.98
Mode	4.67	4	6	3.14	4	1	1	1	2.80
Variance	2.25	2.37	3.01	2.31	2.30	1.93	2.81	2.72	.96
Skewness	−.30	−.06	−.11	−.047	−.09	.41	.19	.20	.51
Kurtosis	−.54	−.76	−.88	−.86	−.38	−.73	−1.14	−1.16	−.01

A K-means cluster analysis was conducted to identify underlying groupings among participants based on eight sub-dimensions: Time, Facilities, Cost (Structural Constraints), Psychological, Lack of Interest, Previous Experience (Intrapersonal Constraints), Lack of Partners, and Lack of Knowledge (Interpersonal Constraints). The analysis specified a three-cluster solution, which was achieved after six iterations. This was determined to be the optimal solution based on cluster centroids, within-cluster sum of squares, and Analysis of variance. Convergence was reached with a maximum absolute change of 0.00 in the cluster centers, indicating a stable solution. The minimum initial distance between cluster centers was 9.05, suggesting sufficient separation among initial centroids.

As illustrated in Table 3, the qualitative characteristics of the final clusters are presented in terms of their mean scores in the sub-dimensions that constitute the structural, intrapersonal, and interpersonal constraints. The final cluster centers revealed distinct patterns across the three groups.

**Figure 1:**
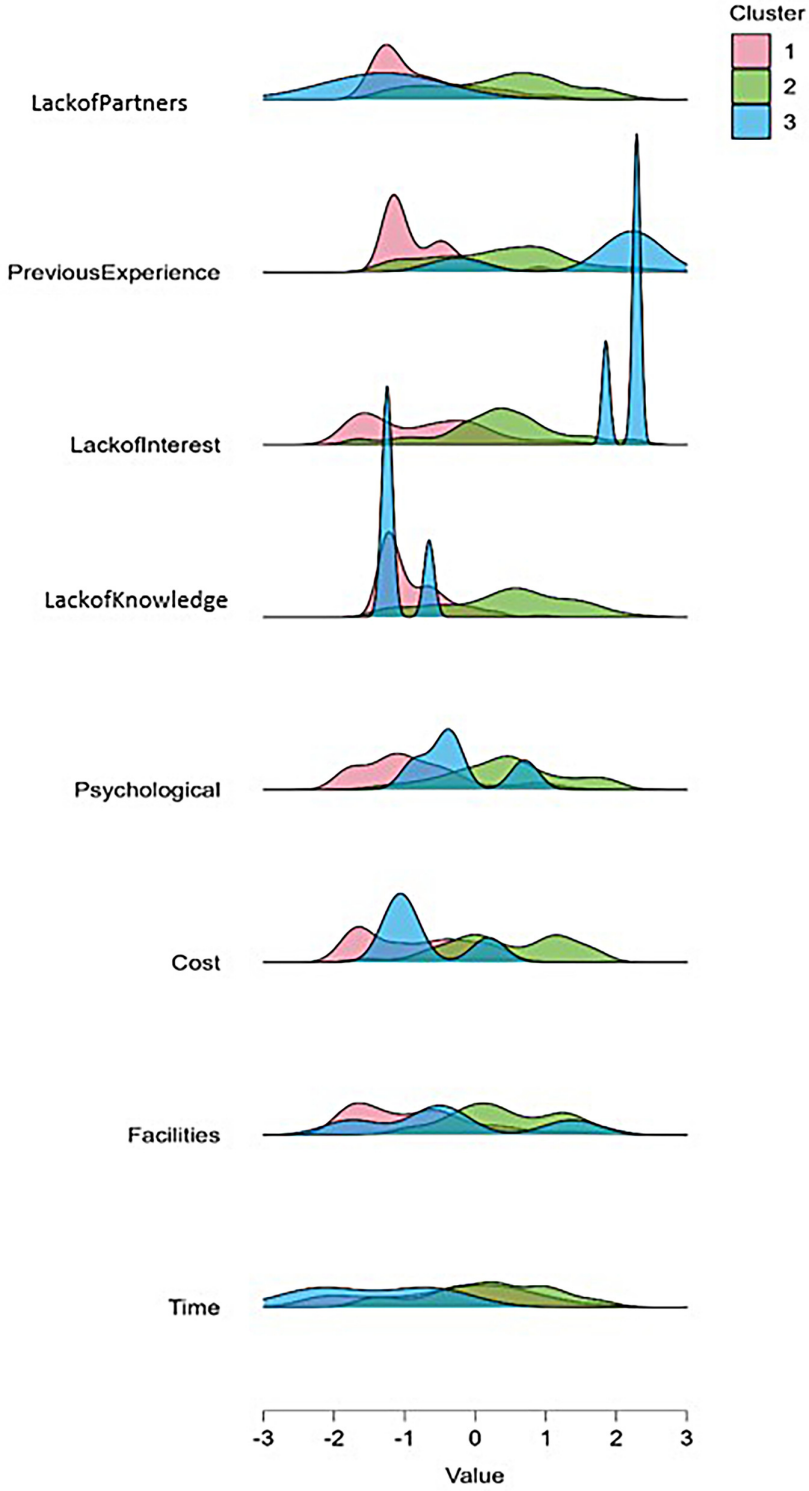
The differentiation of the clusters is based on the structural, intrapersonal, and interpersonal constraints.

• Cluster 1 (*N* = 170) “Medium structural-intrapersonal and high interpersonal constraints” exhibited medium mean scores across most of the dimensions, particularly in Structural Constraints (mean scores in the dimensions of Time, Facilities, Cost), and in Intrapersonal Constraints (mean scores in the dimensions of Psychological, Lack of Interest, Previous Experience). Finally, in terms of the Interpersonal Constraints, the mean scores of Lack of Partners (*M* = 4.72) and Lack of Knowledge (*M* = 4.02) presented a high score.
• Cluster 2 (*N* = 124) “Low structural-intrapersonal and moderate interpersonal constraints” showed low scores across most of the sub-dimensions, particularly in Structural Constraints (sub-dimensions of Time, Facilities, Cost) and in Intrapersonal Constraints (sub-dimensions of Psychological, Lack of Interest, Previous Experience). Concerning Interpersonal Constraints, Lack of Partners presented a medium score (*M* = 2.05) and Lack of Knowledge presented a low score (*M* = 1.56).
• Cluster 3 (*N* = 88) “High structural-intrapersonal and moderate interpersonal constraints” presented high scores across most of the variables, particularly in Structural Constraints (sub-dimensions of Time, Facilities, Cost), and in Intrapersonal Constraints (sub-dimensions of Psychological, Lack of Interest, Previous Experience). Regarding Interpersonal Constraints, the sub-dimensions of Lack of Partners presented a low score (*M* = 2.03), and Lack of Knowledge presented a medium score (*M* = 3.63). The quantitative characteristics are presented in Figure 1.

**Table 3:** Qualitative characteristics of structural, intrapersonal, and interpersonal constraints for the three clusters.

Constraints dimensions		Cluster 1 Medium structural-intrapersonal and high interpersonal constraints	Cluster 2 Low structural-intrapersonal and moderate interpersonal constraints	Cluster 3 High structural-intrapersonal and moderate interpersonal constraints
	Cluster size	*N* = 170	*N* = 124	*N* = 88
Structural constraints	Time	Medium	Low	High
	Facilities	Medium	Low	High
	Cost	Medium	Low	High
Intrapersonal constraints	Psychological	Medium	Low	High
	Lack of interest	Medium	Low	High
	Previous experience	Medium	Low	High
Interpersonal constraints	Lack of PARTNERS	High	Medium	Low
	Lack of knowledge	High	Low	Medium

In order to provide a more detailed description of the cluster differences, one-way ANOVA tests were conducted for each variable. A significant difference was observed among the eight sub-dimensions across the three clusters (*p* < .001). Specifically, the highest F-values were observed for Lack of Partner, F (2, 379) = 324.42, *p* < .001, Lack of Knowledge, F (2, 379) = 153.52, *p* < .001, and Psychological Factors, F (2, 379) = 141.97, *p* < .001, indicating that these variables contributed most strongly to cluster differentiation. Cost, F (2, 379) = 116.63, *p* < .001, Lack of Interest, F (2, 379) = 118.70, *p* < .001, and Previous Experience, F (2, 379) = 115.44, *p* < .001, indicated that these variables contributed a lot to cluster differentiation. Time and Facilities contributed less to cluster differentiation, with F (2, 379) = 21.981, *p* < .001 and F (2, 379) = 73.725, *p* < .001, respectively.

**Figure 2:**
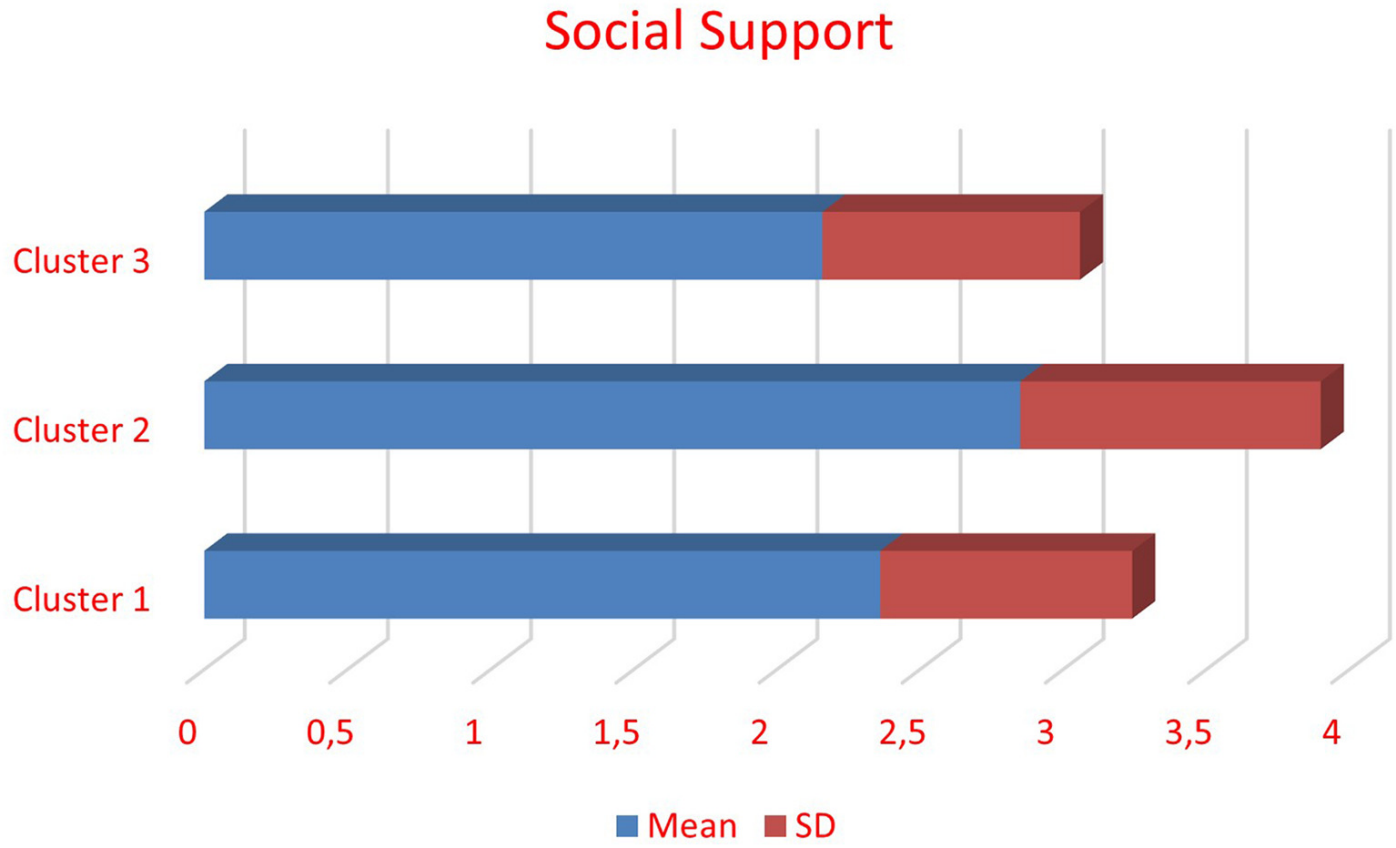
Differences in social support in the three clusters.

An analysis of variance (one-way Anova) was conducted in order to identify whether there are differences among the three clusters and social support, revealing a statistically significant effect, F (2, 379) = 16.05, *p* < .001, *η*^2^ = .08. Bonferroni correction was used as the criterion that the variance was met. Statistically significant differences are identified between Cluster 1, “Medium structural-intrapersonal and high interpersonal constraints”, and Cluster 2, “Low structural-intrapersonal and moderate interpersonal constraints” [*p* < .001, *d* = -.52, CI (-.81, -.23)]. Cluster 2 “Low structural-intrapersonal and moderate interpersonal constraints” (*M* = 2.85, sd = 1.05) presented higher social support than Cluster 1 “Medium structural-intrapersonal and high interpersonal constraints” (*M* = 2.36, sd = .88). Statistically significant differences are also highlighted between Cluster 2 and Cluster 3 “High structural-intrapersonal and moderate interpersonal constraints” [*p* < .001, *d* = .73, CI (.39, 1.08)]. Cluster 2 “Low structural-intrapersonal and moderate interpersonal constraints” (*M* = 2.85, sd = 1.05) presented higher social support than Cluster 3 “High structural-intrapersonal and moderate interpersonal constraints” (*M* = 2.16, sd = .90). Figure 2 represented the information mentioned above.

## 6 Discussion

On conceptualizing the hierarchical model of leisure constraints, Jackson et al. (13) proposed that all individuals face constraints; however, some of them can negotiate and overcome them, and get involved in sports. This model proposed that there are several factors that can act as mediators, interact with constraints, and determine this successful negotiation. Studies so far have proposed and empirically shown that motivation (8, 20), attitudes (3, 9, 21), and personality (63) are among the factors that might interact with constraints and help individuals overcome them. Our study further extends the hierarchical model of leisure constraints, since it proposes that social support can be one of the important factors that determine the successful negotiation of leisure constraints. The results of the current study showed that individuals who had higher social support in their lives were more likely to successfully negotiate leisure constraints and engage in active recreation.

As previously noted, different forms of social support—including instrumental, emotional, and informational support—can facilitate physical activity engagement (34, 35, 41). These three dimensions of social support are particularly applicable in our study to interpret the results. Individuals who receive emotional support are more likely to overcome intrapersonal constraints, since research has shown that intrapersonal constraints are internal ones and they are related to negative self-perceptions about personal abilities, body image, self-esteem, but also a lack of intrinsic motivation (32). This emotional support can come from significant others who can reinforce positive self-perceptions, promote positive feelings, provide empathy, and strengthen their attitudes towards sports. Research, for example, has shown that families who have a strong sport culture are more likely to have their members follow an active lifestyle, due to the creation of an environment that promotes emotional support to their members (25–27). This argument can also be supported based on the social cognitive theory (64), according to which social support can enhance self-efficacy. Increased self-efficacy can lead to overcoming intrapersonal constraints related to low self-perceptions. This emotional support also fits with the definition of the cognitive negotiation strategies, as reported in the study of Jackson and Rucks (60).

In the same way, individuals who receive informational support are more likely to increase their knowledge about opportunities for recreational participation and overcome the “lack of knowledge” related constraints. Previous research has shown that these interpersonal constraints can influence both the preferences for participation but also the selection of individual activities and frequency of participation. Subsequently, they can be either blocking or limiting constraints. This informational support can be informal from the social environment, such as friends, colleagues, etc., but also formal from traditional or new/social media communication channels that sports organizations use today. Considering the increased influence of social media on individuals' behavior (55, 56), it can be argued that social networks and blogs can today have the role of information support agencies for individuals in order to engage in physical activity programs.

The third form of support – instrumental – is also applicable in the current study and can be used to interpret the results. As noted, it is a practical form of assistance, offering tangible solutions to life's challenges (34, 40, 43). This instrumental support can be important for all three types of constraints, but especially for the structural constraints. Friends and the social environment can offer solutions to individuals who want to engage in sports in terms of the selection of the actual activities, advice about sport equipment, cost-related issues, etc. This instrumental element of social support fits with the definition of the behavioral negotiation strategies, as defined by Jackson and Rucks (60).

As previously noted, previous studies had neglected to specifically address the value of social support. In the present study, we investigated in detail the interaction between constraints and social support by examining how the different types of leisure constraints (intrapersonal, interpersonal, and structural) are influenced by social support. We did this by creating profiles of individuals based on the different types of leisure constraints and examining the social support that they receive in their lives. The result of the cluster analysis revealed three distinct groups of individuals, based on their perception of leisure constraints. The first group, which had the highest constraint scores, is particularly influenced by interpersonal constraints. It has been proposed that interpersonal constraints interact with both leisure preferences and actual participation. This cluster includes non-participants or individuals with low sport participation rates. Lack of social networking is therefore the main reason for their low sport participation.

The second cluster is the least constrained one. It had low scores in almost all the constraint dimensions. This group includes individuals who have successfully negotiated constraints and are the most frequent participants. They are probably the most motivated to participate. Previous research has shown that intrinsic motivation can facilitate the successful negotiation of leisure constraints (7, 8).

Finally, the third cluster includes individuals who had low scores in interpersonal constraints and medium to high scores in the rest of the dimensions. The relatively high scores of intrapersonal constraints should be noted. As previously noted, intrapersonal constraints have been proposed to be the most blocking ones in sport participation. The relatively high scores of structural constraints are a noted factor. These constraints can be real or perceived, as Ntovoli et al. (7) discussed.

Comparing the three clusters' scores in social support can lead to some interesting interpretations. The second cluster had the highest scores in social support. This is the cluster with the individuals who reported the lowest level of constraints as well. It can be argued that social support can be one of the reasons for the low constraint scores, extending the hierarchical model of leisure constraint (19) and the negotiation proposition (13). Social support helped these individuals to successfully negotiate and overcome constraints. All three dimensions of social support can play an important role in this successful negotiation. It would, however, be interesting for future research to examine which of the three social support dimensions (instrumental, emotional, and informational) is the most influential in their interaction with leisure constraints. This is an issue for further research. These results propose that sport development officers and policy makers should consider the social aspect of sport and recreation participation when they are developing a promotional plan. Promoting sports within local communities can facilitate social interaction, support, and the overcoming of leisure constraints. The value of community sports has also been emphasized in previous studies (55).

On the other hand, scores in social support were similar in the other two groups. However, the first cluster that reported high scores in interpersonal constraints was the one with the lowest social support scores. As previously noted, interpersonal constraints interact with both preferences and behavior (7–9). They relate to social isolation and the inability of an individual to find partners to participate with. With a lack of social support, it is difficult for individuals to overcome these constraints. The social nature of sport participation should also be noted here (65). Previous studies have shown that group sport participation is an important motivational factor (32). Group participation brings social support in all its forms, but especially in terms of emotional support.

These results have practical implications. They propose that if sport participation is to be increased, the social environment and significant others should play an important supporting positive role. All three elements of social support - instrumental, emotional, and informational – should be targeted and promoted. Communities, families, peers, and colleagues can contribute firstly to emotional support and secondly to instrumental support. It is therefore important from a sport policy perspective to develop educational programs to promote the physical, psychological, and social benefits of sport participation through social groups. Family sport programs, sport programs in corporate/work environments, and community programs can help in this direction. Community leaders can contribute informational support by disseminating information about opportunities to participate in sports and creating social networks. Social media can also play an important role today in informational support. Blogs and social media groups can act as promoters of group exercise and information delivery. This is particularly effective for younger generations (e.g., millennials and Gen Z). All the above strategies can help individuals to overcome certain types of leisure constraints, but especially the intrapersonal ones, which were shown to be the blocking ones. Finally, it must be emphasized that the experience of sport participation is an important factor as well. A positive experience will motivate participants to develop loyalty and foster social support, which will help individuals to overcome constraints. As a closing remark, it should be noted that the promotion of the social aspect of sport participation should be a basic element of the sport policy. Sports have a strong social aspect, and opportunities for socializing should be provided.

## 7 Conclusion

In conclusion, this is the first study that examined the role of social support in overcoming the perception of leisure constraints, using the hierarchical model of leisure constraints and the negotiation proposition. The results showed that social support is one of the factors that interact with leisure constraints. Individuals with high constraint scores, and especially the interpersonal ones, had the lowest social support scores. It can therefore be proposed that social support is one of the factors that determine the successful negotiation of leisure constraints and helps individuals to overcome them. Our study further extends the hierarchical model of leisure constraints and the negotiation proposition by emphasizing the role of the social environment and local communities in motivating individuals to overcome constraints.

## 8 Study limitations and suggestions for future research

As previously noted, the study used a convenient sample of the adult Greek population. Subsequently, the results cannot be generalized. Furthermore, no comparisons between the different demographic groups' scores in constraint and social support scales were made. It is useful in future studies to test, for example, if social support plays a more important role among females, more elderly individuals, or those with a lower educational level. Data from other countries can also help us to understand if culture plays a role in the influence of social support on the perception of constraints.

A final note should be made about the measurement of social support. As previously noted, a one-dimensional scale was used to measure it. As a result, the influence of the different types of social support (e.g., instrumental, emotional, and informational) on leisure constraints was not tested. Finally, it should be noted that the study followed a cross-sectional and not a longitudinal research design. Subsequently, the results do not reveal causal relationships among the variables.

## Data Availability

The original contributions presented in the study are included in the article/Supplementary Material, further inquiries can be directed to the corresponding author.
